# Hospital and Institutional News

**Published:** 1914-07-04

**Authors:** 


### HOSPITAL AND INSTITUTIONAL NEWS.

### THEIR MAJESTIES' VISIT TO GLASGOW.

On Tuesday, July 7, the King and Queen will open the new buildings of the reconstructed Glasgow Royal Infirmary, and will also open the new Royal Hospital for Sick Children at Yorkhill, Glasgow, which replaces the old hospital in Scott Street, which was opened in 1882, and is the only children's hospital in the West of Scotland. The old hospital contained seventy-four beds, which proved inadequate to its requirements. We publish on another page a special plan of the new hospital, together with a criticism of the scheme and a description of the buildings. The new hospital will be opened free from debt, the last £30,000 required to complete the buildings having all been subscribed. Dr. Suttie has been appointed medical superintendent, and the work in the new buildings will begin almost immediately.

### THE GLASGOW ROYAL INFIRMARY.

The rebuilding of this institution was begun nine years ago, the foundation-stone of the surgical block, which was the first portion to be started in July 1905, having been laid by their Majesties, then Prince and Princess of Wales, in April 1907. The history of the reconstruction is interesting. In 1897, the then Lord Provost of Glasgow, Sir David Richmond, promoted a memorial of the Diamond Jubilee of Queen Victoria, the original object of which was to rebuild the front block of the Royal Infirmary. An executive committee of prominent citizens was formed to raise from £80,000 to £100,000. This executive committee ultimately enlarged their scheme by obtaining plans for the reconstruction of all the ward blocks, which were adopted and submitted to the managers of the infirmary. The managers felt, however, that much more was necessary to complete and efficiently equip a great infirmary, and they elaborated a complete scheme, involving a total expenditure of £500,000, under which the reconstructed Royal Infirmary would accommodate 660 patients. The plans were drawn up by Mr. James Miller, A.R.S.A., F.R.I.B.A., and were subsequently remitted for revision, in so far as the internal arrangements were concerned, to Sir Henry C. Burdett and Dr. D. J. Mackintosh, M.V.O., to examine and make suggestions as they might think advisable for their improvement in detail or as a working whole, with the proviso that the general ground plan of the main ward blocks should be adhered to, and the number of beds not diminished. The reconstructed infirmary buildings are the result of the co-operation of these three gentlemen; the plans represent a modern up-to-date hospital and contain several novel features of interest. We hope to publish them shortly.

### RADIUM FOR MANCHESTER.

On June 23 a meeting was held in Manchester, convened by the Lord Mayor, to stimulate a public appeal for funds to provide a central supply of radium for the use of hospitals in Manchester. The medical aspects of the case were presented by Dr. Dawson Turner, of Edinburgh, who contended that radium had passed the experimental stage in medicine. This bold assertion was, in our judgment, exceedingly unwise and inaccurate. That a doctor should have been imported from Edinburgh to address this meeting and to pronounce this dogma makes one wonder whether the reason may have been that no Manchester medical man could be found who would commit himself so optimistically. Dr. Turner, we are pleased to be able to add, would not describe radium as a cure for cancer; but hoped that with more experience and larger quantities of radium, still better results would be obtained than heretofore. This passage, obviously, is in flat contradiction to that to which exception has been taken above; for clearly he foreshadows further experimentation with a view to improving the results of treatment. At any rate, Sir Ernest Rutherford, who also spoke, said that the Manchester University wants at least a gramme of radium at once, and two or three times as much in a year or two. Contracts have already been signed for the delivery of two-fifths of a gramme at a cost of £8,000. We are constrained to repeat our view that radium treatment is still in an experimental stage, probably quite an early one at that; and that institutions proposing to spend many thousands of pounds on procuring this substance should clearly comprehend the risk that in a few years its use may possibly be abandoned altogether; though on the other hand it is equally possible that it may prove the sovereign cure that enthusiasts hope for.

### MANCHESTER'S CENTRAL ACCIDENT HOSPITAL.

When the removal of the Manchester Royal Infirmary to its new site was agreed upon a few years ago, a condition was made that a central accident branch should be erected in the heart of the city. The sum of £25,000 was asked for to build the accident hospital, and it is hoped that the last £5,000 of this sum will be subscribed before the opening, which is to take place in about a month's time. It should be obvious enough that all those who favoured the removal of the infirmary should appreciate the fact that its necessary corollary is a central branch to which accidents in streets, warehouses, factories, and mills can be taken. These, and others, may indeed realise that this central branch in Manchester is only part of the new movement for taking away hospitals from centres of traffic to, at all events, less crowded parts of the towns, by which the hospital itself becomes a part merely of an organisation which has its beds as near to fresh air and quiet as possible, its consultative department for out-patients, and its central department for accident cases. The removal scheme in Manchester cannot be regarded as complete until the accident branch is as firmly established as the infirmary itself.

### SIR A. L. JONES MEMORIAL HOSPITAL.

The late Sir Alfred Jones did so much for institutions and for medical science that it is good to know that his bequest for a hospital at Garston is bearing fruit, and that the new Accident and Emergency Hospital there will bear his name. When Garston was incorporated with Liverpool, ten years ago, there was (and still is) a small accident hospital supported out of the rates. The Liverpool City Council proposed to abolish it, but the Garston folk objected, on the ground that it was one of the liabilities taken over by the city; the hospital accordingly remained a charge upon the rates, but the needs of the district have far outgrown its resources. Sir Alfred Jones lived within the old Garston boundaries, and left £10,000 for a new hospital. As this would relieve the Liverpool rates of an annual charge of £600, Garston appealed to the Corporation for a grant in aid equal to this sum capitalised. This large sum Liverpool declined to consider, but after much argument was persuaded to contribute £7,500. This quite generous treatment Garston still affects to regard as niggardly and unjust; but at all events the new hospital is to be proceeded with. A central site has been obtained, and the demolition of some schools and old cottages upon it is already begun. The old Garston Bridewell is also on the site, and it is pleasing that this is to be preserved. The new hospital is to be known as the Sir Alfred L. Jones Memorial Hospital. Captain E. W. Turner is the chairman of the Hospital Committee; Dr. W. B. Paterson and Mr. A. Robertson are the secretaries; Mr. A. E. Jacob is chairman of the Finance Sub-Committee; and Mr. J. E. Tinnè is chairman of the Sites and Building Committee.

### PERSONAL SERVICE IN NORTH STAFFORDSHIRE.

A striking instance of the right sort of spirit in the financial administration of a voluntary hospital is forthcoming from North Staffordshire. As chairman of the Subscription Committee—the mere existence of such an original body proves the alertness of the management—Colonel A. H. Heath is trying to raise the number of subscribers to the North Staffordshire Infirmary to five thousand. So he persuades local charitable committees to convene meetings, at which he pleads his cause, eloquently and convincingly. At Talke, for example, he recently addressed the local Hospital Saturday Committee at a meeting held in the Council school. While acknowledging the great value of Hospital Saturday collections, he explained to the gathering the needs of the infirmary for a larger subscription list, as well as for various improvements which cannot at present be contemplated for want of funds. He enlarged also upon the ever-increasing work done by the infirmary, and upon the expensive alterations and additions which have been lately undertaken there: the new operating theatre and pathological department. Finally, he suggested that a committee should be appointed at Talke to canvass for annual subscriptions to the infirmary, and stated that all subscriptions of five shillings and upwards would be acknowledged in the annual report. This proposition, having been seconded by the rector of the parish, was carried unanimously, and the committee aforesaid was forthwith appointed. This is just the kind of energy which might, with advantage, be infused into the management of other voluntary hospitals. The North Staffordshire Infirmary has evidently picked the right man in Colonel Heath for the chairmanship of the Subscription Committee, and we trust that this example of personal service and of good organisation will find many imitators.

### THE JURISDICTION OF THE CHARITY COMMISSIONERS.

A decision of very great importance to hospital trustees and to the philanthropic public has recently been arrived at in the Chancery Division, touching the right of the Charity Commissioners to supervise the deeds and accounts of endowment of the Richard Murray Hospital at Consett. To put matters briefly, Mr. Murray conveyed to trustees in 1911 certain real property, stocks, shares, and £10,000 in cash to found this institution; his whole intention by this course being to avoid the interference and authority of the Charity Commission. A site was selected and bought, buildings planned and begun, and the institution was to be ready for opening in August of this year. In November 1913, however, the Charity Commissioners called upon the trustees to furnish particulars of their trust—in other words, they claimed to exercise jurisdiction over the charity. The trustees, believing themselves protected from this interference, refused to obey the Commissioners, who accordingly issued a writ of attachment against them. Mr. Justice Joyce has upheld this writ; we must assume that as the law stands he could do no otherwise, for he appears to have done it most reluctantly, and to have described the action of the Charity Commission as "ridiculous." It is certainly astonishing that a private individual, endowing a charity during his lifetime with the express object of reserving it from the control of this body, should be unable to do so, and should expose his trustees to very serious pains and penalties. A correspondent of the *Yorkshire Post* very aptly remarks that if donors are to have their wishes disregarded in this fashion gifts to charity will necessarily stop, as such proceedings do not encourage generosity. Considering that the trustees were carrying out the express wishes of the patron, it is certainly hard on both parties to have their arrangements upset in this high-handed manner.

### MEDICAL REPRESENTATION UPON THE LONDON INSURANCE COMMITTEE.

The names of five candidates who have been duly nominated for election as medical representatives upon the Insurance Committee of the County of London have been published by the Commission, and ballot papers distributed. In alphabetical order the five are: R. V. Donnellan, L.R.C.P., L.F.P.S.G., a Lewisham practitioner who is a divisional surgeon to the police and also out-patient surgeon at the Miller Hospital and Royal Kent Dispensary; Major Greenwood, M.R.C.S., L.R.C.P., D.P.H., of Hackney, editor of the *Poor Law Medical Officer*, public vaccinator, medical officer to the Post Office, and district surgeon to the City of London Lying-in Hospital; D. B. King, M.D., M.R.C.P., Mayfair, assistant physician to the Royal Hospital for Diseases of the Chest; W. H. F. Oxley, M.R.C.S., L.R.C.P., Poplar, district medical officer to several shipping companies; and Alfred Welply, M.D., formerly of Ilford and now of Finsbury. It will be noticed that four of the competing five are general practitioners, perhaps of no very special distinction, but at any rate of substance. It is also noteworthy that only two of the five were educated professionally in London, and that neither of these two has a University degree. Two of the other three candidates come from Scotland, and one from Ireland. The election does not seem to be creating any interest among the members of the medical profession in London, the great majority of whom will probably boycott it altogether by declining to vote.

### PARLIAMENTARY GRANTS FOR FEEDING SCHOOL CHILDREN.

Subject to certain conditions laid down in a Board of Education circular just issued, a grant will be made during the current financial year towards the cost of meals for children attending elementary schools. The grants, if Parliament agrees to provide the money, will be made this financial year in respect of work done during the year ending March 31, 1914. The aggregate amount of the grants will be limited, and they will be distributed at the discretion of the Board. The following points will be taken into consideration in assessing the grants: (*a*) the extent to which the work is co-ordinated with that of the School Medical Service; (*b*) the care exercised in the selection of the children for admission to the meals; (*c*) the sufficiency and suitability of the dietary; (*d*) the extent to which attention is given to the educational aspect of the work; (*e*) the suitability of the accommodation and equipment and the efficiency of the service and supervision of the meals; (*f*) the completeness of the arrangements made for ascertaining and recording the effect of the meals on the physical and mental condition of the children; (*g*) the economical administration of the work. The Board recognise that the work in respect of which these grants will be assessed has been wholly done before the issue of any regulations; and hope on this occasion to be able to award a grant of 50 per cent. wherever the service is found to have been reasonably satisfactory.

### DR. SALTER AND THE LONDON INSURANCE COMMITTEE.

The King's Bench has upheld the claim of Dr. A. Salter, of Bermondsey, to be reinstated on the panel list of the County of London under the National Insurance Act. This practitioner was formerly on the panel, but was struck off it by the London Committee because he declined to comply with sundry regulations made by the latter. His objection to these was that they required him to convert himself into a clerk in order to save trouble to the panel chemists; so he refused to obey them. He was accordingly struck off the list, but it appeared to Mr. Justice Bray that in this action the Committee had exceeded its legal powers. Dr. Salter had done nothing which entitled the Committee to dismiss him without inquiry and without due notice. Failing these, he was entitled to remain on the panel, and the Committee will have to reinstate him thereon, unless, of course, the decision is reversed on appeal. Dr. Salter is well known in the world of politics; he is not of the most docile type of practitioner, and the Committee will probably think twice before risking any further litigation with him. He had a brilliant career as a student, and did some years' work at bacteriology before settling down in private practice.

### SCARLET FEVER IN THE METROPOLIS.

The epidemic of scarlet fever in London continues to increase. At the last meeting of the managers of the Metropolitan Asylums Board the number of patients reported under care for this disease was 3,030, compared with 2,957 a fortnight previously. In order to cope with these large totals the Hospitals Committee of the Board have been obliged to suspend temporarily the admission of cases of measles and of whooping cough to the Board's fever hospitals. At the date of the report in question, scarlet fever accounted for two-thirds of the cases under treatment in those institutions. It was also reported to the managers that the number of inmates of casual wards has continued to decrease, and that the Casual Wards' Committee have decided in consequence to close wards at Hackney, Holborn, Chelsea, Paddington, and Camberwell for varying periods during the holiday season, and in two cases until further notice. The scheme for providing homeless persons found in the streets in Central London at night has met with such satisfactory results as to lead to its extension. Police constables now provide homeless persons in different districts with tickets directing them to proceed to an office, where they are given orders of admission either for a shelter belonging to a casual agency or for a casual ward.

### THE BRITISH HOSPITAL FOR MOTHERS.

Some particulars of the British Hospital for Mothers and Babies are given in a County Council report, the new scheme having been submitted by the Charity Commissioners to the London County Council. The scheme provides for the amalgamation of the British Lying-in Hospital—which used to be in Endell Street, Covent Garden—with the Home for Mothers and Babies in Wood Street, Woolwich, under the new title mentioned. The objects of the consolidated charity are the establishment and maintenance in Woolwich of a maternity hospital and school for the higher training of midwives, the cost to be defrayed as far as possible out of contributions and grants to the trustees, the balance being taken from the funds of the charity, which amount approximately to £12,400 in cash and investments. The Local Government Committee of the London County Council is calling the attention of the Charity Commissioners to the fact that although there do not appear to have been any restrictions in regard to the area of benefit of the British Lying-in Hospital, it no doubt served the central districts of London, that the amalgamation with the Woolwich Home will probably shift the area of benefit to the district of Woolwich, which is on the outskirts of the county, and that it may follow that persons resident outside the county will participate in the benefits to a greater extent than at present. Provision is to be made for the complete training of district midwives at the lowest practicable fees, and for the reception and training of midwifery pupils.

### WALSALL WORKPEOPLE AND THEIR HOSPITAL.

The loyal support which many provincial hospitals, particularly in the Midlands and the North of England, receive from the industrial and other workers who benefit by the treatment provided has often been the subject of comment in The Hospital. An excellent example of the right way to go about gaining and keeping such support is provided by the managers of the Walsall and District Hospital. An annual inspection of the hospital by representatives of the workpeople of the town takes place. This year June 13 was fixed for this inspection, which brought large numbers of people to the hospital. After tea had been served in an empty ward the chairman of the hospital (Mr. Cotterell) addressed the representatives, and explained the work which was being done and the work that could be done if more money were obtained. Last year the workpeople's collections brought in £1,600, and Mr. Cotterell hopes to see this sum raised to £2,000, or even £3,000. He was able to inform the assembly that the empty ward in which they were meeting would not be available for the purpose next year. It had been closed for want of funds; but a public-spirited lady in Walsall (Mrs. Hill) offered to maintain one cot for a year if others would guarantee the cost of the other cots. A special sub-committee was appointed, and the result was that the £400 a year required to open the ward was promised. Since works collections were instituted in Walsall this hospital has benefited to the extent of £18,000: a fine record, easily comprehensible when the loyal recognition of the workers' interest and help implied by this annual inspection is borne in mind.

### THE FINANCES OF THE MACCLESFIELD INFIRMARY.

The governors of the Macclesfield General Infirmary have unanimously carried a motion, proposed by Mr. Walter Brown, for the appointment of a special committee to investigate the financial position of the institution. From the admirably lucid and common-sense speech of the mover of the resolution it is evident that this step has not been taken any too soon. During the last six years expenditure has exceeded income by £2,125. Last year the income was £3,229, and the expenditure £3,817, the deficit of £588 being the largest of the series. At the beginning of this year the infirmary owed £1,558 at the bank, a debt which may very likely be larger still by the beginning of 1915. Mr. Brown thought it improbable that economies could be introduced without impairing efficiency; he testified to the careful management and the absence of waste. He laid stress upon the shrinkage in income. Endowments, which have not been touched during these six lean years, bring in £2,280 a year; but subscriptions of individuals have fallen from £531 to £404, and the workpeople's collections are down from £300 to £190. In a town of 35,000 inhabitants only 170 subscribe to the infirmary. We congratulate the governors on taking steps to secure better support, and upon their courage in refusing to sell investments; and do not doubt that with energy and personal service the new committee will soon be able to better the financial situation of their hospital. A legacy of £300 from Miss Brocklehurst was announced, and is to be used to reduce the indebtedness to the bank.

### THE GREAT YARMOUTH GENERAL HOSPITAL.

In view of Sir Henry Burdett's Report on this institution, which appeared in The Hospital of December 13 last, we are glad to note that at the annual meeting of the governors, which was held in the last week of June, it was shown that the income has increased by upwards of £500 during 1914 compared with 1913. Electric light has been introduced throughout the hospital, the Nurses' Home has been enlarged, and the committee have had under consideration the improvement of the income of the hospital, and have appointed a special Finance Sub-Committee to investigate the position. As a result a large committee of ladies has been organised, and a special effort will be made during the summer to obtain subscriptions during one whole week, to be known in future as Hospital Week. We hope that the remaining £1,000 a year of additional income which this hospital requires will speedily be forthcoming. We regret to notice, however, that the annual report contains no intimation that the committee have taken any steps whatever to secure the better and fuller equipment of the ward kitchens and ward adjuncts. The Report to which we have referred pointed out that such matters urgently required a prompt remedy. We would remind the committee that an adequate number of cupboards for the reception of linen and other stores is greatly needed; that the lavatories are crowded with all sorts of things which ought never to find a place there, to the great discomfort of the in-patients; and that there is a lack of adequate and hygienic lockers and of chairs throughout the wards. Sir Henry Burdett found that the equipment of this hospital was starved, especially in the older portions of the buildings, and we hope the local Press will keep these needs before the committee and the people of Yarmouth until an effective remedy is found and applied.

### A NEW HOSPITAL CALLED FOR.

The Beccles Hospital was established in 1874 at Beccles in Suffolk with fifteen beds. At the annual meeting in June attention was called to the committee's desire that a new hospital should be erected, as the present buildings have been condemned as out of date and unsuitable. No adequate scheme of reconstruction is possible, and there must be a new building on a new site, the estimated cost of which is from £6,000 to £7,000. The Mayor's recommendation was heartily endorsed by Dr. Wood-Hill, a member of the staff, who was unanimously in favour of a new hospital. Dr. Fox, supporting the scheme, declared the present operation theatre to be a disgrace to the town. The project was unanimously endorsed, and the Mayor undertook to call a public meeting and give the proposal for a new hospital energetic support.

### NEW O.-P. DEPARTMENT AT THE DERBYSHIRE CHILDREN'S HOSPITAL.

The new out-patient department of the Derbyshire Children's Hospital, which has cost £1,750, was opened on June 24 by Mrs. Walter Evans. An anonymous donor has provided £1,000 towards this sum, but the remaining £750 is still required. There is also a deficiency of annual income amounting to about £500 which requires wiping out. The new building is a great advance upon the old one, which had been quite outgrown by the needs of the institution. Included in it are an *x*-ray installation, a dark room, an operating theatre, and a recovery room, besides the usual examination rooms. There are also a new waiting-room and dispensary. The chairman of the board of management, Mr. F. W. Greaves, has been associated with this hospital ever since its establishment in 1877. At that time he was honorary auditor, a position which in 1890 he exchanged for that of treasurer; since 1903 he has been chairman, and his services were eulogised in flattering terms by speakers at the opening ceremony of this new department. We note also that a representative of the working-men's fund (Mr. Munn), who seconded the vote of thanks to Mrs. Evans, hoped that the time would come when every works in Derby would contribute to this admirable institution.

### THE CHELSEA WOMEN'S HOSPITAL SCHEME.

The recent laying of the foundation-stone of the new buildings of the Chelsea Hospital for Women, on a site generously given by Lord Cadogan, bordering on Arthur Street, marks another stage in the steady progress of this scheme. Thanks to Lord Cadogan, Mr. T. Dyer Edwards, and to the trustees of the Zunz Bequest, the sum required has been reduced to £16,000. Lord Cadogan went so far also as to imply that his generous intentions were not exhausted, and that his wish was to see the completion of the new development. Lord Cadogan's memories go back to the time when the institution was originally founded; and he can recall each step in the changes that have led to Mr. Keith Young's and Mr. Hall's plans for the new buildings, which comprise the main building containing accommodation for seventy-six patients, a theatre unit and an out-patient department, the detached nurses' home, and a pathological block. The present buildings have already done thirty-three years' service.

### THE VOLUNTARY AID DETACHMENTS.

An exceedingly strong committee has been appointed by the War Office to inquire into the working and organisation of the Voluntary Aid Detachments of the Territorial Force. This intelligence will be welcome to all who have taken interest in the progress and development of the Territorial Force or in that of Red Cross work in this country. As has been over and over again emphasised in these columns, the present method of expecting men and women to enrol themselves in Voluntary Aid Detachments in order to fill gaps deliberately left in the organisation of the Territorial Force, to train themselves in the manifold duties of their work, and to subscribe among themselves or their friends the funds necessary for purchasing the equipment essential to make their training efficient, is a request so extraordinary that in no other country but this would there be the faintest chance of any response to it. The terms of reference of the committee are as follows: "To inquire into and report upon the difficulties which have been experienced in co-ordinating the work of the societies and associations in forming, registering, training, administering, and controlling Voluntary Aid Detachments, and to make suggestions for amending the existing schemes for the organisation of voluntary aid, with a view to the removal of such difficulties."

### ANOTHER "ROYAL" COT AND AN "ARMY" COT.

To the collected list of Royal cots in various hospitals given in these columns a few weeks ago (The Hospital, May 23, p. 221; and June 6, p. 272) must now be added the latest instance. The "Children's Salon"—an organisation founded by the *Gentlewoman* twenty-four years ago—has just endowed its eleventh cot. This cot is in the Edinburgh Hospital for Women and Children, and Her Majesty the Queen, who was asked to name it, has expressed the wish that it should be called after the Prince of Wales. This is the second cot of the eleven which the Queen has named; the first one is in the Great Ormond Street Hospital, and was named the "Princess Mary Victoria" cot in 1903, for the lifetime of Princess Mary of Wales, as she then was. We note also that Prince Alexander of Teck is endeavouring to raise £1,000 to endow an "Army" cot in the Royal Waterloo Hospital for Women and Children, towards which £500 has already been subscribed. The cot is to be reserved for the children of discharged soldiers resident in London.

### THE NEW SUPERINTENDENT OF MONTROSE ROYAL ASYLUM.

At the special meeting of the Board of this Asylum held on June 24, Dr. Charles John Shaw, the Medical Superintendent of the Argyll and Bute District Asylum, out of a number of candidates, was unanimously appointed Medical Superintendent at Montrose with a salary of £1,000, with free house, light, vegetables, etc. Dr. Shaw graduated at Edinburgh in 1902, and during his undergraduate career showed marked talent in mental diseases, in which he gained distinction at the final examinations. In 1906 he obtained the M.D. degree, and was awarded a gold medal for his thesis on the liability of the insane to tuberculosis. He is also a Fellow of the Royal College of Physicians, Edinburgh. He has devoted himself exclusively to the study of mental disease and the care of the insane in asylums. He has held the appointments of assistant to Dr. Lewis C. Bruce at the Perth District Asylum, Murthly; senior assistant physician of the Montrose Royal Asylum; and senior assistant physician of Glasgow Royal Asylum, Gartnavel, the largest asylum for the reception of private patients in Scotland. In 1908 Dr. Shaw was appointed Medical Superintendent of the Argyll and Bute District Asylum. Dr. Shaw's distinguished career makes his latest appointment full of promise, and we shall expect to hear of him and his work as he makes still further progress on the road to the highest distinction.

### THE METROPOLITAN HOSPITAL SATURDAY FUND.

The Hospital Saturday Fund Journal for June contains some interesting facts which show that the receipts in London for the first five months of 1914 exceed those of 1913 by upwards of £700, the net increase approaching £500, or £100 a month. We extract the following brief abstract of the benefits conferred by the Hospital Saturday Fund, in addition to the support it renders to the hospitals: "During the first half of the current year sanatorium benefit was granted to ninety patients. When insured persons are subscribers to this Fund, the Insurance Committees pay the whole of the cost. The total sum received at the office during the first half of 1914 exceeds £300. Non-insured persons pay about one-third of the cost, and the other two-thirds are paid by the Fund. The number of Convalescent Home benefits issued during the half-year amounted to 863; about one-fifth of these had free letters, and the other four-fifths paid about 6s. per week. The number of surgical appliance benefits issued was 3,916, the patients paying half the cost. The dental work of the Fund is increasing rapidly. During the past six months 2,076 cases were considered, and grants were made in 1,957 cases, the grants amounting to over £1,144. The ambulance benefits granted are also appreciated, especially the removing of patients to or from hospitals." We congratulate the committee and Mr. A. W. Davis, the secretary, on these excellent results. The committee and officers have waged a brave fight for many months, and are now able to record that the Fund is recovering from the set-back caused by the operations of the National Insurance Act, through the hearty co-operation of employers and employed, upon whose support the finance and prosperity of the Fund must depend.

### HOSPITAL COLLECTION ON A RACECOURSE.

A most praiseworthy effort was recently made by the Working Men's Committee in aid of the Horley Cottage Hospital. The committee, which was only formed at the beginning of the present year, had already collected by weekly subscriptions the sum of £40 in three months, but it was resolved that a special effort should be made by way of a Hospital Saturday Collection on Saturday, June 20, when, with the co-operation of many ladies who provided stalls of real flowers, in addition to the Alexandra roses, the sum of £105 8s. 5d. was obtained. The most noteworthy feature of the collection was, perhaps, the special permission granted to several of the collectors to visit Gatwick Racecourse. The school authorities of Reigate and Red Hill evidently and rightly believe that practical interest in hospital work is a necessary part of true and patriotic citizenship, for the secretary of the Reigate and Red Hill Hospital has recently received the sum of £8 7s. 5d. from the children of the Borough Elementary Schools, being the result of a collection made among the scholars on Empire Day.

### THIS WEEK'S DRUG MARKET.

The outstanding feature of the drug market this week is the inactivity of business; with the exception of small orders covering immediate requirements, little interest is being taken in the market, but there is nothing particularly unusual in this state of affairs at this season of the year. The demand for citric and tartaric acids, and other "seasonable goods," is, of course, well maintained, and if the warm weather continues prices of both the articles named may be expected to advance. Cod-liver oil is naturally very quiet, and the tendency of prices is downwards rather than upwards; the Norwegian cod-fishing season is practically over, and the final results show that the yield of cod-liver oil has been considerably larger than that of last season; under these circumstances there appears to be little likelihood of values being advanced to any material extent. Cream of tartar has an upward tendency in price, and the advanced quotations for essence of lemon are maintained. A further advance in the price of santonin may take place at any moment, notwithstanding that the value of this drug has already reached an extraordinary figure. Opium has an upward tendency in price, and the same may be said of morphine, while codeine is dearer. Lower prices are expected for attar of roses in consequence of a much better crop in Bulgaria. Cape aloes is somewhat lower in price, as also is ipecacuanha. The expected rise in the price of quinine has not yet taken place, but in some quarters it is believed that it will shortly be announced.

